# Curcumin Reduced H_2_O_2_- and G2385R-LRRK2-Induced Neurodegeneration

**DOI:** 10.3389/fnagi.2021.754956

**Published:** 2021-10-15

**Authors:** Jinru Zhang, Kai Li, Xiaobo Wang, Amber M. Smith, Bo Ning, Zhaohui Liu, Chunfeng Liu, Christopher A. Ross, Wanli W. Smith

**Affiliations:** ^1^Department of Neurology and Clinical Research Center of Neurological Disease, The Second Affiliated Hospital of Soochow University, Suzhou, China; ^2^Department of Psychiatry and Behavioral Sciences, Johns Hopkins University School of Medicine, Baltimore, MD, United States; ^3^Department of Human Anatomy and Cytoneurobiology, School of Biology and Basic Medical Sciences, Soochow University, Suzhou, China; ^4^Institute of Neuroscience, Soochow University, Suzhou, China

**Keywords:** LRRK2-G2385R, curcumin, oxidative stress, ROS, H_2_O_2_–hydrogen peroxide, Parkinson's disease

## Abstract

Mutations in *leucine-rich repeat kinase 2 gene* (*LRRK2*) are the most frequent genetic factors contributing to Parkinson's disease (PD). G2385R-*LRRK2* increases the risk for PD susceptibility in the Chinese population. However, the pathological role of G2385R-*LRRK2* is not clear. In this study, we investigate the roles of G2385R-LRRK2 in neurodegeneration underlying PD pathogenesis using cell biology and pharmacology approaches. We demonstrated that expression of G2385R-LRRK2-induced neurotoxicity in human neuroblastoma SH-SY5Y and mouse primary neurons. G2385R-LRRK2 increased mitochondrial ROS, activates caspase-3/7, and increased PARP cleavage, resulting in neurotoxicity. Treatment with curcumin (an antioxidant) significantly protected against G2385R-LRRK2-induced neurodegeneration by reducing mitochondrial ROS, caspase-3/7 activation, and PARP cleavage. We also found that the cellular environmental stressor, H_2_O_2_ significantly promotes both WT-LRRK2- and G2385R-LRRK2-induced neurotoxicity by increasing mitochondrial ROS, caspase-3/7 activation, and PARP cleavage, while curcumin attenuated this combined neurotoxicity. These findings not only provide a novel understanding of G2385R roles in neurodegeneration and environment interaction but also provide a pharmacological approach for intervention for G2385R-LRRK2-linked PD.

## Introduction

Parkinson's disease (PD) is the second most common neurodegenerative disease with movement disorders. The clinical symptoms include bradykinesia, myotonia, and rest tremor, and the pathological features include loss of dopaminergic neurons in the substantia nigra and the deposition of protein aggregates, lewy bodies (Kalia and Lang, 2015). Mutations in *leucine-rich repeat kinase 2 gene* (*LRRK2*) are recognized as the most frequent genetic factors contributing to familial and sporadic PD. The proportion of *LRRK2* gene mutations in autosomal-dominant PD patients is about 5–15%, and in sporadic PD patients it is 1–3% (Healy et al., 2008). According to the Human Gene Mutation Database, more than 100 *LRRK2* variants have been reported to be associated with PD (http://www.hgmd.cf.ac.uk/ac/index.php). Among those variants, G2019S is the most frequently reported mutation in the Caucasian population, but it is rare in the Asian population (Clark et al., 2006; Healy et al., 2008; Lee et al., 2017). Genetic studies indicate that about 5.5% of sporadic PD patients carried the G2385R (Xie et al., 2014) variant in the Asian population. G2385R increases the risk for PD susceptibility by two to three folds (Healy et al., 2008; Zhang et al., 2018). However, the neuropathological roles of G2385R-LRRK2 are not fully understood.

LRRK2 is a multiple-domain protein containing a kinase domain, a GTPase domain, and a WD40 domain (Liu et al., 2016). LRRK2 possesses both kinase and GTPase activities. Most PD-linked LRRK2 mutations (e.g., G2019S) create an increase in kinase and GTPase activities, which play critical roles in PD pathogenesis (Liu et al., 2011a). Unlike the G2019S mutation that is located in its kinase domain, G2385R is located in the WD40 domain. As the WD40 domain binds with several vesicle-associated proteins, the G2385R variant correlates with a reduced binding affinity of LRRK2 to synaptic vesicles (Piccoli et al., 2014). G2385R-LRRK2 protein localize to the cytoplasm and form aggregates in transfected HEK293 cells (Tan et al., 2007). Previous studies suggest that G2385R does not affect LRRK2 kinase activity (West et al., 2007; Nichols et al., 2010; Tan et al., 2010), or may slightly reduce the kinase activity (Ho et al., 2016; Carrion et al., 2017). Therefore, G2385R may affect cellular functions via a different pathway than other PD-linked mutations in PD pathogenesis.

In this study, we are interested in understanding the roles of G2385R in affecting the neurodegeneration underlying PD pathogenesis using cell biology and pharmacology approaches. We found that overexpression of G2385R-LRRK2 induced neurotoxicity via an increase in oxidative stress, thereby activating the apoptotic pathway, and that curcumin (an antioxidant) significantly protected against G2385R-LRRK2-induced neurotoxicity. These findings provide novel insight into the mechanisms of G2385R-LRRK2-linked neurodegeneration and a potential treatment strategy for PD patients harboring G2385R.

## Materials and Methods

### Reagents

Antibodies against PARP (CST, 9532) and cleaved-PARP (CST, 9541) were from Cell Signaling, Inc. The anti-flag antibody, H_2_O_2_, and curcumin were from Sigma. Anti-LRRK2 [MJFF4 (c81-8)] was from MJFF. ECL goat anti-Rabbit IgG and ECL goat anti-Mouse IgG were from GE Healthcare (NA934V and NA931V, respectively). Mito-tracker and MitoSox were from Molecular Probe.

### Plasmids, Cell Culture, and Transfection

WT-LRRK2, G2385R-LRRK2, and G2019S-LRRK2 constructs contain human LRRK2 cDNA with Flag-tags as described previously (Yang et al., 2012; Thomas et al., 2016). Human embryonic kidney (HEK293) cells were from the American Type Culture Collection (ATCC; Manassas, VA, USA) and were grown in Dulbecco's modified Eagle medium (DMEM; Gibco) with 10% heat-inactive fetal bovine serum (FBS; Gibco) and 1% penicillin–streptomycin (Gibco) in a humidified 5% CO_2_ atmosphere at 37°C. SH-SY5Y human neuroblastoma cells were from ATCC grown in Opti-Mem I media with 10% FBS and 1% penicillin-streptomycin. Cells were transfected with vectors, GFP, and various LRRK2 constructs using LipofectAMINE and a Plus reagent (Invitrogen, Carlsbad, CA) according to the manufacturer's protocol. For treatment experiments, curcumin (1 μM) was added 1 h prior to transfection and then transfected various constructs in transfection media without curcumin. Post-transfection lasted 4 h, and cells were incubated to normal medial with curcumin (1 μM in double-distilled H_2_O) for 48 h. H_2_O_2_ (100 μM) was added 4 h post-transfection and until the end of the experiment.

### Cell Viability Assays

SH-SY5Y cell viability assays were performed as described previously with slight modification (Smith et al., 2005; Yang et al., 2012; Thomas et al., 2016). Briefly, post-transfection lasted 46 h, and neurons expressing GFP were imaged for 2 h with 20 min intervals on a Zeiss Axiovert 200 inverted microscope. The healthy GFP-positive cells were counted (where at least one process is smooth with two folds of cell body length) by an automatic program to detect healthy and smooth neurites and DAPI nuclei as described previously (Smith et al., 2005; Yang et al., 2012).

### Mitochondrial Reactive Oxygen Species Assays

Cells were transfected with vector or various LRRK2 constructs for 12–48 h. MitoSOX (5 μM) and MitoTracker (1 μM) were added 20 min before imaging as described previously (Wang et al., 2020). The unused free MitoSox was washed away using PBS. Hoechst 33342 (blue) was used for nuclei staining. Fluorescent images were captured under a fluorescent microscopy image system (Carl Zeiss) with identical exposure settings. NIH image software was used to quantify the density of MitoSOX (Red), which represents the levels of mitochondrial superoxide. The MitoTracker was used to confirm the localization of MitoSOX in mitochondria.

### Caspase-3/7 Activity Assay

The Nexcelom ViaStain™ Live Caspase-3/7 Detection kit (CSK-V0003-1, Nexcelom) was employed to detect caspase-3/7 activities according to manufactural protocol. The assay is based on the NucViewTM reagent with a nucleic-acid-binding dye linked to a fluorescent probe that is attached to a four-amino-acid peptide sequence DEVD (Asp-Glu-Val-Asp) to form a cell-membrane-permeable DEVD-DNA complex. During apoptosis, activated caspase-3/7 cleaves the DEVD-DNA dye complex to release the high-affinity DNA dye, which translocates to the nucleus and binds to the DNA, producing a bright green, fluorescent signal. When cells are healthy, the nucleic-acid dye is linked to the DEVD peptide sequence, and the dye is unable to bind to DNA, resulting in a lack of flourescence. Cells were transfected with a vector or LRRK2 constructs for 48 h and then incubated with NucViewTM reagent for 30 min. The green fluorescence was read using a Celigo fluorescent reader.

### Primary Cortical Neurons Injury Assay

Mouse primary cortical neurons were generated from E16 embryos as described previously (Nucifora et al., 2016), and they were cultured in Neurobasal medium with 2 mM Glutamax, 2% B27, and 1% PenStrep antibiotics. At 5 days *in vitro* (DIV), neurons were co-transfected with various LRRK2 plasmids and green fluorescent protein (GFP) at a ratio of 10:1 using Lipofectamine 2000 (Invitrogen) as described previously (16, 17). About 95% of GFP-positive cells expressed LRRK2 variants confirmed by co-immunostaining using anti-GFP and anti-LRRK2 antibodies. Curcumin was added 1 h prior to transfection, and H_2_O_2_ (100 μM) was added 4 h post-transfection.

Neuronal injury assays were performed as described previously (Smith et al., 2005; Liu et al., 2016; Wang et al., 2021). Neurons were imaged at end of experiments. Neurons with injury neurites were blindly quantified using NIH ImageJ software. Injured neurons were defined as displaying loss of neuronal projections, a disappearance of nerites, the presence of soma fragmentation, nerites with beading morphology, or a soma shape change from oval to completely circular. An arbitrary morphological score was assigned as either 100 for healthy cells or 0 for injured cells in each frame. The mean morphological score of all the cells was calculated. Data analysis was performed using a Gehan-Breslow test to determine the statistical difference between groups, followed by an All Pairwise Multiple Comparison (Holm-Sidak method) to identify the differences between groups.

### Western Blot Analysis

Cells were collected using cell lysis buffer (Cell Signaling, Inc, CST-9803S) with 20 mM Tris-HCl (pH 7.5), 150 mM NaCl, 1 mM Na_2_EDTA, 1 mM EGTA, 1% Triton, 2.5 mM sodium pyrophosphate, 1 mM beta-glycerophosphate, 1 mM Na_3_VO_4_, 1 μg/ml leupeptin, and 1 mM PMSF. Cell lysates from each group were subjected to protein assays. An aliquot of 30 μg protein from each sample was resolved on 4–12% NuPAGE Bis/Tris gels and transferred onto polyvinylidene difluoride membranes (Millipore). The membranes were blocked with 4% milk for 1 h, then incubated with various primary antibodies as indicated in Reagents section: anti-Flag (1:1,000 dilution), rabbit anti-PARP (1:1,000 dilution), and rabbit anti-cleaved PARP (1:1,000 dilution) followed by HRP-conjugated secondary antibodies: goat anti-Rabbit IgG (1:5,000 dilution) or goat anti-Mouse IgG (1:5,000 dilution). Protein bands were detected by ECL enhanced chemiluminescence reagents (PerkinElmer) as described previously (Li et al., 2014; Yang et al., 2018; Arbez et al., 2020).

### Statistical Analysis

Quantitative data represent the means ± SEM of three separate experiments. There were three parallel reactions for each sample for all biochemistry experiments. Statistically significant differences among groups were analyzed by ANOVA by using the GraphPad Prism 5 software. The *p* value for significance was 0.05.

## Results

### G2385R-LRRK2 Induced Toxicity in SH-SY5Y Cells

**Figure 1:**
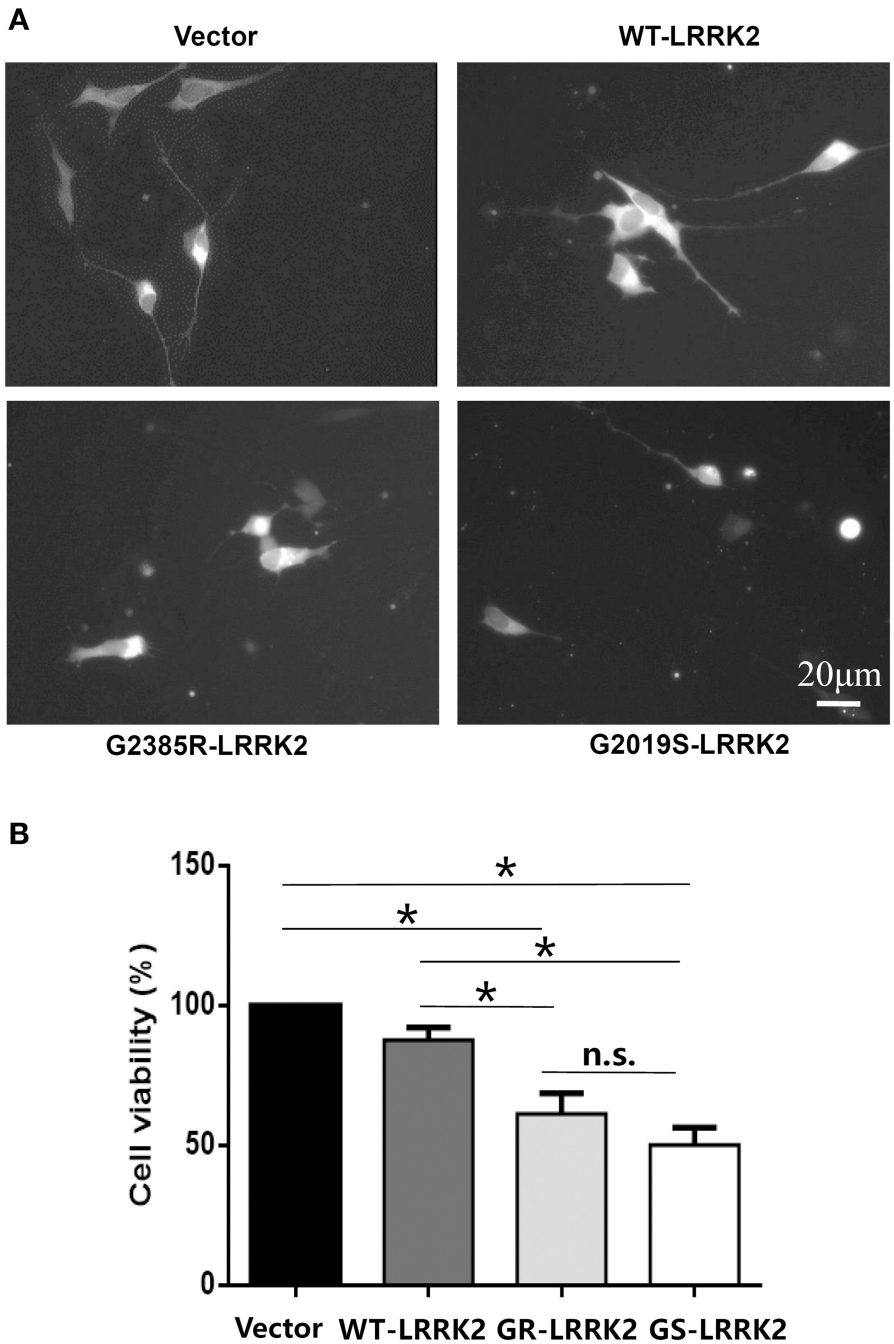
G2385R(GR)-LRRK2 induced neurodegeneration in SH-SY5Y cells. SH-SY5Y cells were co-transfected with GFP and vector or various LRRK2 constructs at 1:10 ration for 48 h. **(A)** Representative fluorescent images of cells in each group. **(B)** Cells viability. *p < 0.05, statistical significance between groups as indicated. NS, no significance; GS, G2019S.

G2385R-LRRK2 was widely identified in the genetic studies of the Asian population (Zhang et al., 2018). Its neurobiological function is understudied compared with the most common PD-linked mutation, G2019S-LRRK2. We found that transient expression of G2385R-LRRK2 significantly induced neurotoxicity in human neuroblastoma SH-SY5Y cells compared with vector cells alone or cells expressing wild-type (WT)-LRRK2 (Figure 1). Compared to G2019S-LRRK2, G2385R-LRRK2 induced slightly less cell toxicity but exhibited no statistical difference. Thus, we only focused on G2385R-LRRK2 in this study.

### G2385R-LRRK2 Increased the ROS Level in Mitochondria and Activated Caspase 3/7

**Figure 2:**
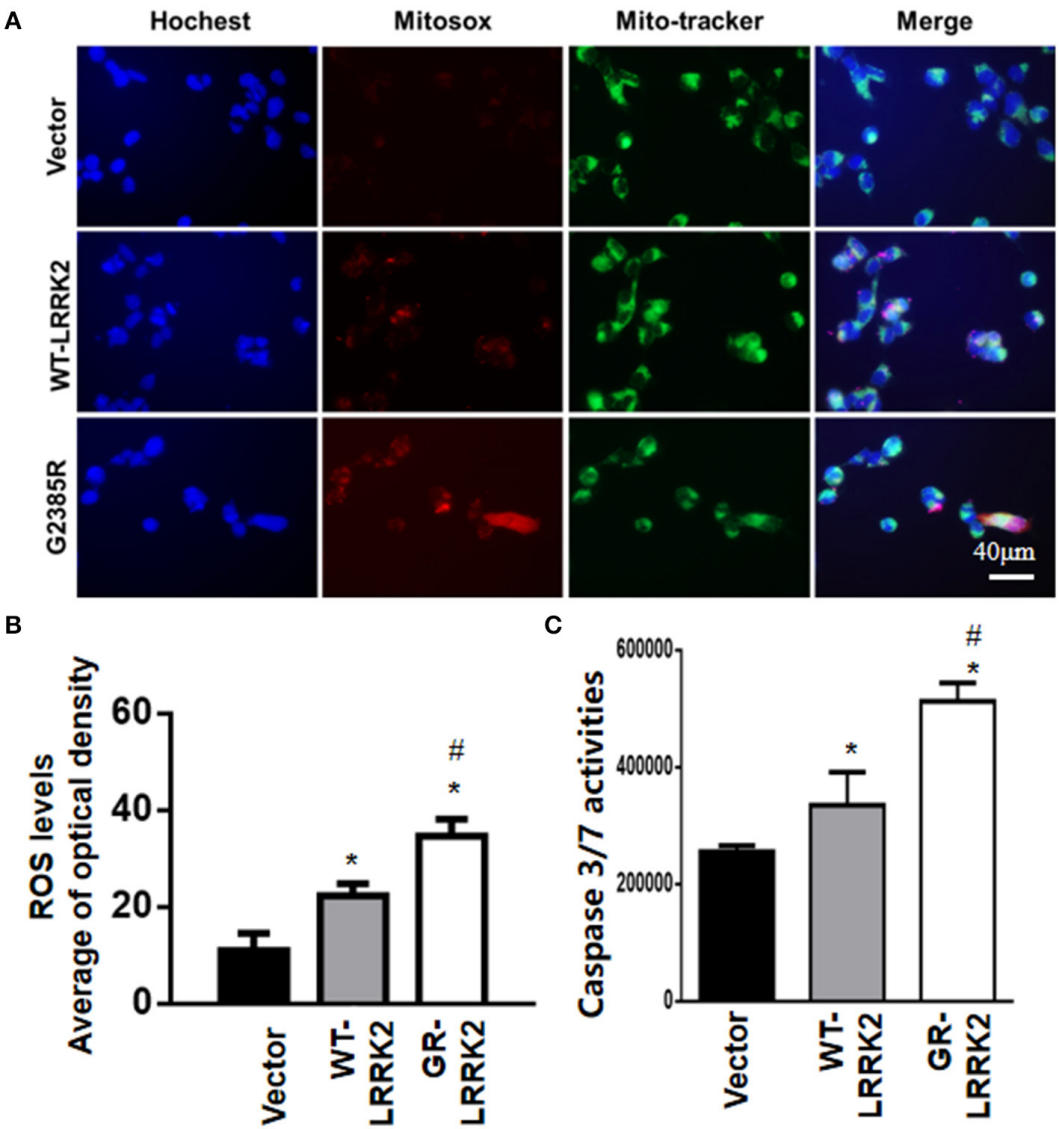
G2385R-LRRK2 significantly increased mitochondrial ROS and caspase-3/7 activation. SH-SY5Y cells were transfected with vector or various LRRK2 constructs as indicated for 12–48 h. MitoTracker (green) and MitoSOX (red) reagents were added to cells 5 min before taking pictures. **(A)** Representative cell images at 12-h post transfection with MitoSOX. **(B)** Relative density of MitoSOX (red) fluorescence in each group at 12-h post transfection. **(C)** The lysate of cells expressing vector or various LRRK2 variants for 48 h were subjected to caspase-3/7 activity assays. *p* < 0.05 by ANOVA, * vs. cells expressing vector. ^#^ vs. cells expressing WT-LRRK2.

**Figure 3:**
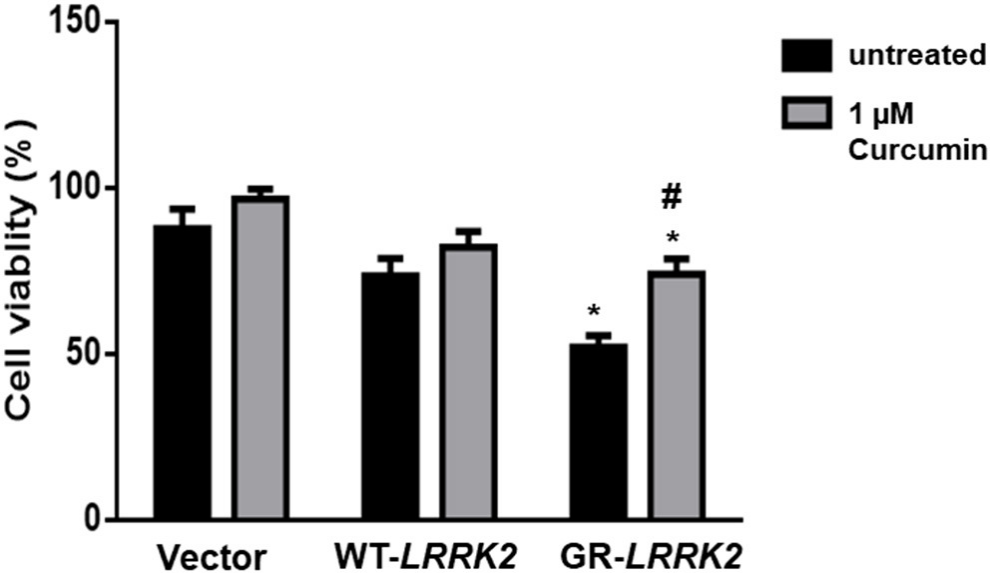
Curcumin protects against G2385R-LRRK2-induced neurotoxicity. Cells were transfected with vector or various LRRK2 constructs as indicated. Curcumin (1 μM) was added 1 h before transfection, and then 4-h post transfection till the end of the experiment. Cell viability was measured 48-h post-transfection. * *p* < 0.05, * vs. cells expressing vector. ^#^ vs. cells expressing G2385R-LRRK2.

To study whether G2385R-LRRK2 alters mitochondrial ROS, MitoSOX florescent assays were used. The MitoSOX™ Red reagent can selectively label the production of mitochondrial superoxide under fluorescence microscopy. Expression of either WT or G2385R-LRRK2 increased the ROS level in mitochondria compared with vector cells (Figures 2A,B), while cells expressing G2385R-LRRK2 had more mitochondrial ROS than those of WT-LRRK2. Increased ROS (oxidative stress) could induce mitochondrial dysfunction and result in the activation of a caspase cascade. In fact, we found that expression of WT-LRRK2 slightly increased caspase 3/7 activities (Figure 2C). However, G2385R-LRRK2 significantly increased caspase 3/7 activities compared with vector cells or cells expressing WT-LRRK2. Treatment with an antioxidant, curcumin, at 1 μM concentration (the most effective dose based on our pilot experiment) significantly protected against G2385R-induced neurodegeneration in SH-SY5Y cells compared with vehicle control (Figure 3).

### H_2_O_2_ Promotes G2385R-LRRK2-Induced Neurodegeneration in Primary Neurons

**Figure 4:**
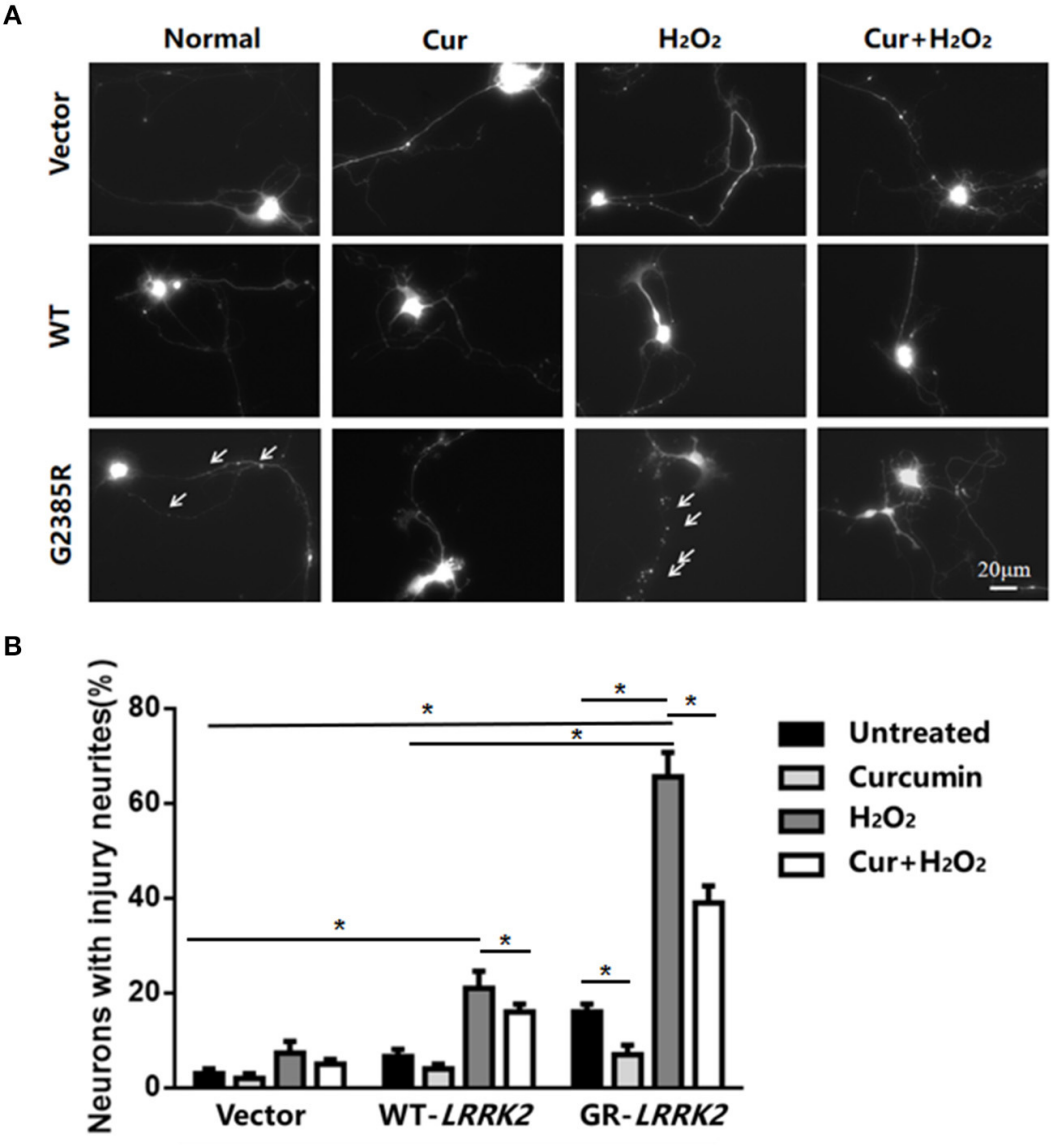
Curcumin protects against combined neurotoxicity of G2385R-LRRK2 and subtoxic H_2_O_2_. Mouse cortical primary neurons were co-transfected with GFP and vector or various LRRK2 constructs at 1:10 ratio, with or without a subtoxic dose of H_2_O_2_ exposure and curcumin (1 μM). Neurons with injured neurites were measured. **(A)** Representative fluorescent images of neurons in each group. **(B)** Neurons with injury (white arrow) were counted in each group. * *p* < 0.05, statistical significance between groups as indicated.

To further validate G2385R-LRRK2-induced neurodegeneration, we used mouse primary cortical neurons. Consistent with our findings in SH-SY5Y cells, expression of G2385R-LRRK2 dramatically induced neurite injury in mouse primary neurons compared with vector or WT-LRRK2 groups (Figure 4). Curcumin significantly attenuated G2385R-induced neurite injury compared with the vehicle group (Figure 4).

To further study the interaction between environmental stress and G2385R mutation, we added subtoxic doses of H_2_O_2_ (100 μM) in neurons expressing vector or LRRK2 (WT or G2385R) variants. We found that H_2_O_2_ promoted both WT-LRRK2 and G2385R-LRRK2-induced neurite injury (Figure 4), but the neurons with G2385R were more vulnerable to H_2_O_2_ than those of WT-LRRK2 cells. There was about a 3.5-fold increase in neurons with neurite injury in the G2385R-LRRK2 plus H_2_O_2_ group compared to vector cells with H_2_O_2_ (Figure 4). Curcumin also protected against this combined neurotoxicity (H_2_O_2_ and G2385R), up to 50% (Figure 4).

### Curcumin Attenuated H_2_O_2_ and G2385R-LRRK2-Induced Mitochondrial ROS and Caspase-3/7 Activation

**Figure 5:**
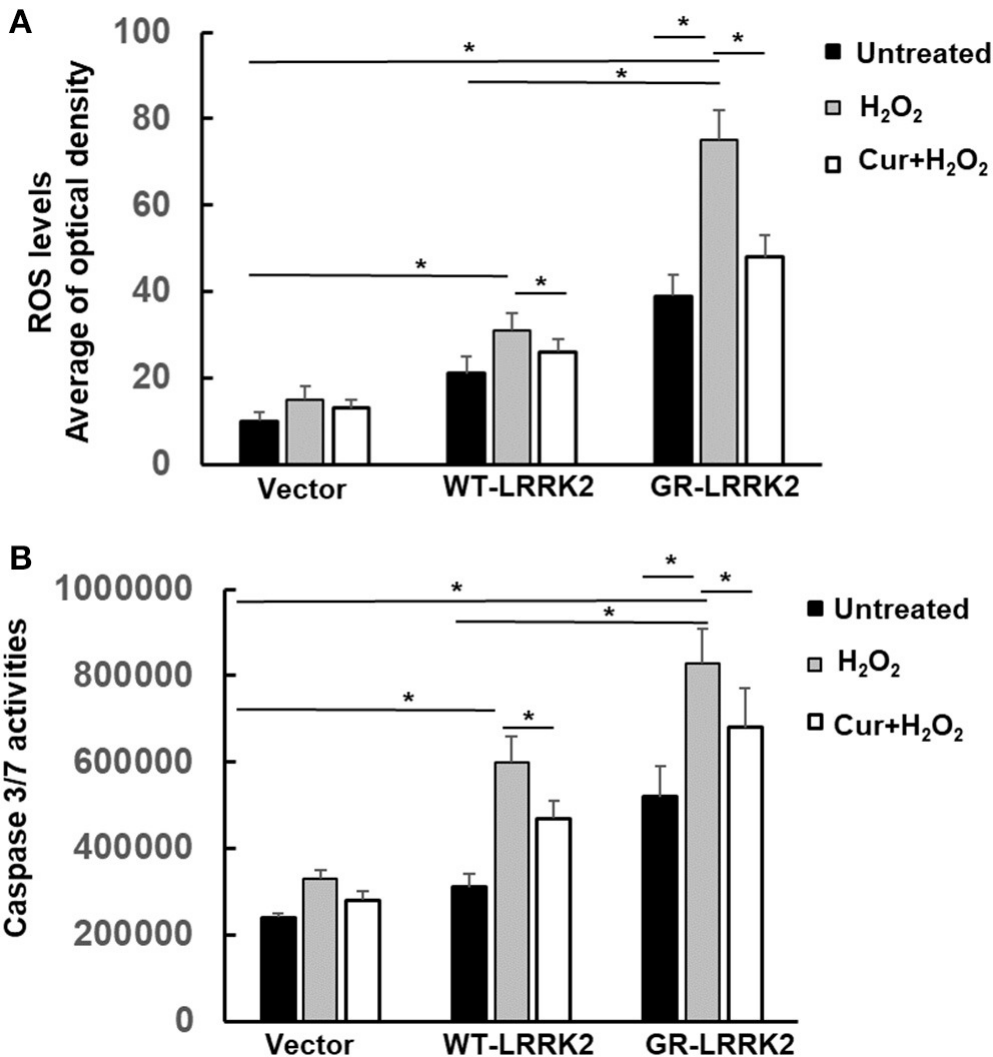
Curcumin reduced H_2_O_2_ and G2385R-LRRK2-induced mitochondrial ROS and caspase-3/7 activation. SH-SY5Y cells were co-transfected with vector or various LRRK2 constructs with or without a subtoxic dose of H_2_O_2_ exposure and curcumin (1 μM) for 12–48 h. **(A)** Mitochondrial ROS in each group was measured at 12 h after treatment of H_2_O_2_ and curcumin. **(B)** Caspase-3/7 activities were measured at 48 h after treatment of H_2_O_2_ and curcumin. * *p* < 0.05, statistical significance between groups as indicated.

Exposure of subtoxic doses of H_2_O_2_ (100 μM) in SH-SY5Y cells also dramatically increased both WT-LRRK2- and G2385R-LRRK2-induced mitochondrial ROS levels compared to no exposure control group (Figure 5A), while the ROS increased more in cells expressing the G2385R variant. Moreover, Curcumin treatment significantly attenuated the mitochondrial ROS induced by H_2_O_2_ and G2385R-LRRK2 compared with vehicle control (Figure 5A). Exposure of subtoxic doses of H_2_O_2_ also dramatically increased both WT-LRRK2 and G2385R-LRRK2-induced caspase-3/7 activation compared to those cells without H_2_O_2_ (Figure 5B). Curcumin treatment significantly reduced caspase-3/7 activation (Figure 5B).

### Curcumin Attenuated PARP Cleavage Induced by H_2_O_2_ and G2385R-LRRK2

**Figure 6:**
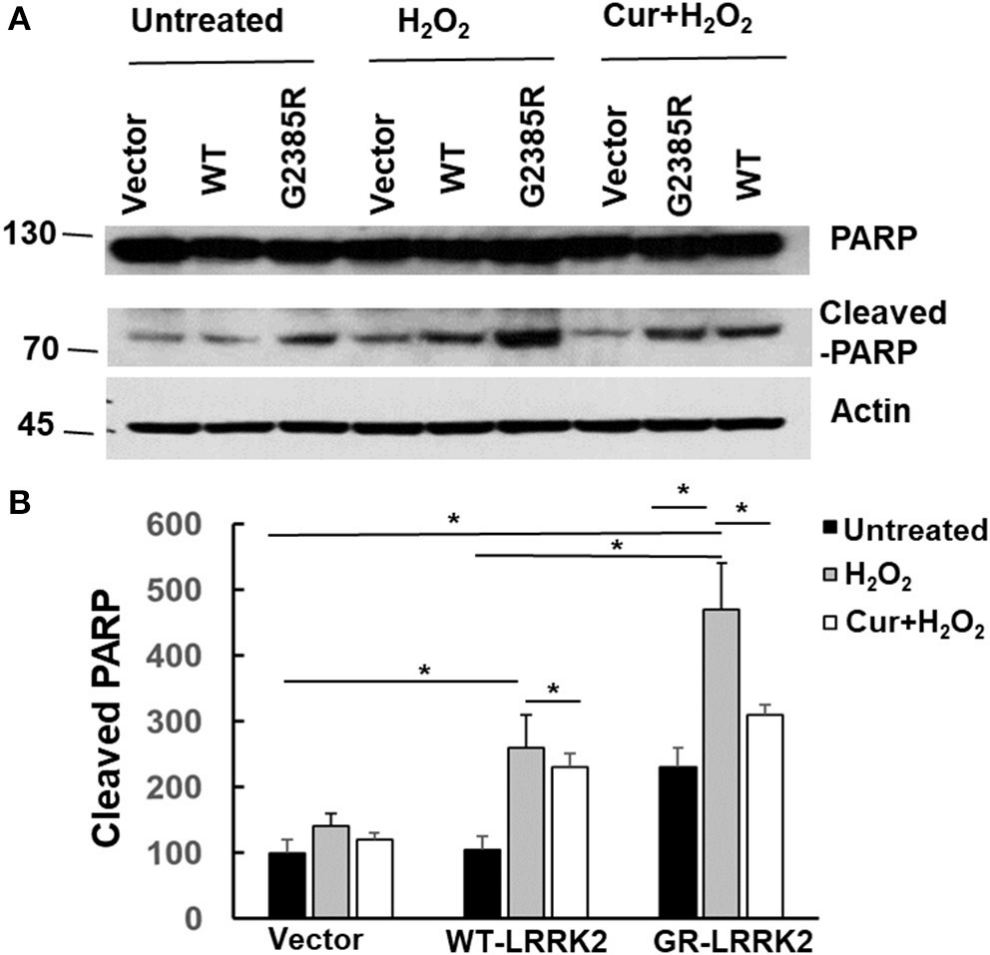
Curcumin reduced H_2_O_2_ and G2385R-LRRK2-induced PARP cleavage. HEK293 cells were co-transfected with vector or various LRRK2 constructs with or without a subtoxic dose of H_2_O_2_ exposure and curcumin (1 μM) for 48 h. Cell lysates were subjected to western blot analysis using antibodies against PARP, cleavage PARP, and actin. **(A)** Representative blot from three separated experiments. **(B)** Quantification of **(A)** * *p* < 0.05, statistical significance between groups as indicated.

PARP is an enzyme in the nucleus and can be cleaved (inactive) by active caspase-3 during apoptosis. To assess whether G2385R alters PARP cleavage, we used HEK293 cells transiently transfected with LRRK2 constructs as these cells have high transfected efficiency (over 80%) and extract the protein, then perform western blot analysis. We found that expression of mutant G2385R-LRRK2 significantly increased PARP cleavage compared to vector cells (Figure 6). Moreover, H_2_O_2_ dramatically increased G2385R-LRRK2-induced PARP cleavage compared to those cells without H_2_O_2_ (Figure 6). While curcumin treatment significantly attenuated PARP cleavage-induced by H_2_O_2_ and G2385R-LRRK2 compared with vehicle controls (Figure 6).

## Discussion

In this study, we demonstrated that expression of G2385R-LRRK2 induced neurodegeneration in human neuroblastoma SH-SY5Y and mouse primary neurons. G2385R-LRRK2 increased mitochondrial ROS, activated caspase-3/7, and increased PARP cleavage, resulting in neurotoxicity. Treatment with curcumin (an antioxidant) significantly protected against G2385R-LRRK2-induced neurodegeneration by reducing mitochondrial ROS, caspase activation, and PARP cleavage. We also found that the cellular environmental stressor, H_2_O_2_, significantly promotes G2385R-induced neurotoxicity by increasing mitochondrial ROS, caspase-3/7 activation, and PARP cleavage, while curcumin attenuated this combined neurotoxicity.

G2385R-LRRK2 variant is associated with an increased risk of PD in the Chinese population, and subjects harboring this variant were ~2.5 times more likely to develop PD (Healy et al., 2008; Zhang et al., 2018). G2385R is in the WD40 domain, which is involved in a variety of cellar functions such as signal transduction, pre-mRNA processing, and cytoskeleton assembly. The G residue is hydrophobic, while R has a positive charge and is hydrophilic. G2385R alters the motifs from 2,385 to 2,390 (GLidCV), a potential N-myristoylation site of LRRK2 protein, which has implications in posttranslational N-myristoylation related to apoptotic events (Zha et al., 2000; Vilas et al., 2006). As is consistent with the structural and genetic analysis, we found that expression of G2385R-LRRK2 significantly reduced cell viability in SH-SY5Y cells and increase neurite injury in the primary neurons due to an increase in mitochondrial ROS, activation of caspase 3/7, and cleavage of PARP, which are key events in apoptosis.

Oxidative stress plays a critical role in aging and neurodegenerative diseases including PD. Oxidative stress has been reported in various cellular and animal PD models and post-mortem human PD specimens (Abou-Sleiman et al., 2006). We and others previously found that PD risk mutant alleles (e.g., A53T alpha-synuclein, G2019S-LRRK2) causes more severe neurotoxicity in the presence of oxidative stress environment (Smith et al., 2005; West et al., 2007; Li et al., 2010; Liu et al., 2011b). The generation of ROS within neuronal cells can trigger stress-linked signaling pathway, resulting in the activation of a caspase cascade and apoptosis. In this study, we found that subtoxic H_2_O_2_ did not alter vector control cells but significantly potentiated both WT-LRRK2 and G2385R-LRRK2-induced neurotoxicity by an increase in mitochondrial ROS, caspase-3/7 activation, and PARP cleavage. Cells with G2385R-LRRK2 are more vulnerable than cells with WT-LRRK2. This is consistent with previous studies that oxidative stress promotes G2385R-LRRK2-induced cell death and protein aggregation in HEK293 cells (Tan et al., 2007). Together, these findings indicate that G2385R alters LRRK2 function and triggers ROS generation and caspase/PARP-linked apoptotic pathways.

Curcumin is a potent antioxidant (Zhao et al., 1989; Gupta et al., 2011; Liu et al., 2011b). The free radical scavenging effect of curcumin is several times higher than that of vitamin E (Zhao et al., 1989). Previous studies have shown that curcumin can be protected against neurodegeneration in various PD models, including reduction of the damage of 1-methyl-4-phenyl-1,2,3,6-tetrahydropyridine (MPTP) and 6-hydroxydopamine (6-OHDA), G2019S-LRRK2 on dopaminergic neurons (Rajeswari, 2006; Wang et al., 2009). Our results showed that curcumin dramatically attenuated G2385R-induced neurodegeneration and further protected against the combined neurotoxicity induced by both H_2_O_2_ and G2385R. The protective effect of curcumin is through reduction the mitochondrial ROS, caspase-3/7 activation, and PARP cleavage. Curcumin is a food additive with a large safety window. There are no adverse or toxic effects with doses from 2,000 to 8,000 mg/day according to clinical studies (Srimal and Dhawan, 1973; Hsu and Cheng, 2007). Curcumin protects against neuronal degeneration in other *in vitro* and *in vivo* models of neurodegenerative diseases (e.g., Alzheimer's disease) as well. Thus, curcumin could be a potential prevention or treatment agent for G2385R-LRRK2-linked PD or related disorders.

In conclusion, our findings demonstrated that G2385R-LRRK2 induced neurodegeneration and H_2_O_2_ significantly potentiated G2385R neurotoxicity in cultured SH-SY5Y cells and primary neurons. Curcumin protected against combined neurotoxicity of G2385R-LRRK2 and H_2_O_2_ via reducing mitochondrial ROS, caspase-3/7 activation, and PARP cleavage. These findings not only provide a novel understanding of G2385R roles in neurodegeneration and environment interaction but also provide a pharmacological approach for intervention for G2385R-LRRK2-linked PD.

## Data Availability Statement

The original contributions presented in the study are included in the article, further inquiries can be directed to the corresponding author.

## Author Contributions

JZ, CL, and WS: conception and design of the study. JZ, KL, XW, AS, BN, ZL, CL, CR, and WS: acquisition, analysis, and interpretation of data. JZ and WS: drafting the manuscript and statistical analysis. All authors contributed to the article and approved the submitted version.

## Funding

This work is supported by NIH Grants: R01NS093383, R01NS119208, and R01NS120879 to WS.

## Conflict of Interest

The authors declare that the research was conducted in the absence of any commercial or financial relationships that could be construed as a potential conflict of interest.

## Publisher's Note

All claims expressed in this article are solely those of the authors and do not necessarily represent those of their affiliated organizations, or those of the publisher, the editors and the reviewers. Any product that may be evaluated in this article, or claim that may be made by its manufacturer, is not guaranteed or endorsed by the publisher.
